# Supplementary material for: Conserved strategies of RNA polymerase I hibernation and activation
Source: Nat Commun. 2021 Feb 3;12:758. doi: 10.1038/s41467-021-21031-8 (PMC7859239; doi:10.1038/s41467-021-21031-8)
Supplement: Supplementary file 1 — Supplementary Information [file 41467_2021_21031_MOESM1_ESM.pdf]

Supplementary Information

Conserved strategies of RNA polymerase I hibernation and activation

Heiss et al.

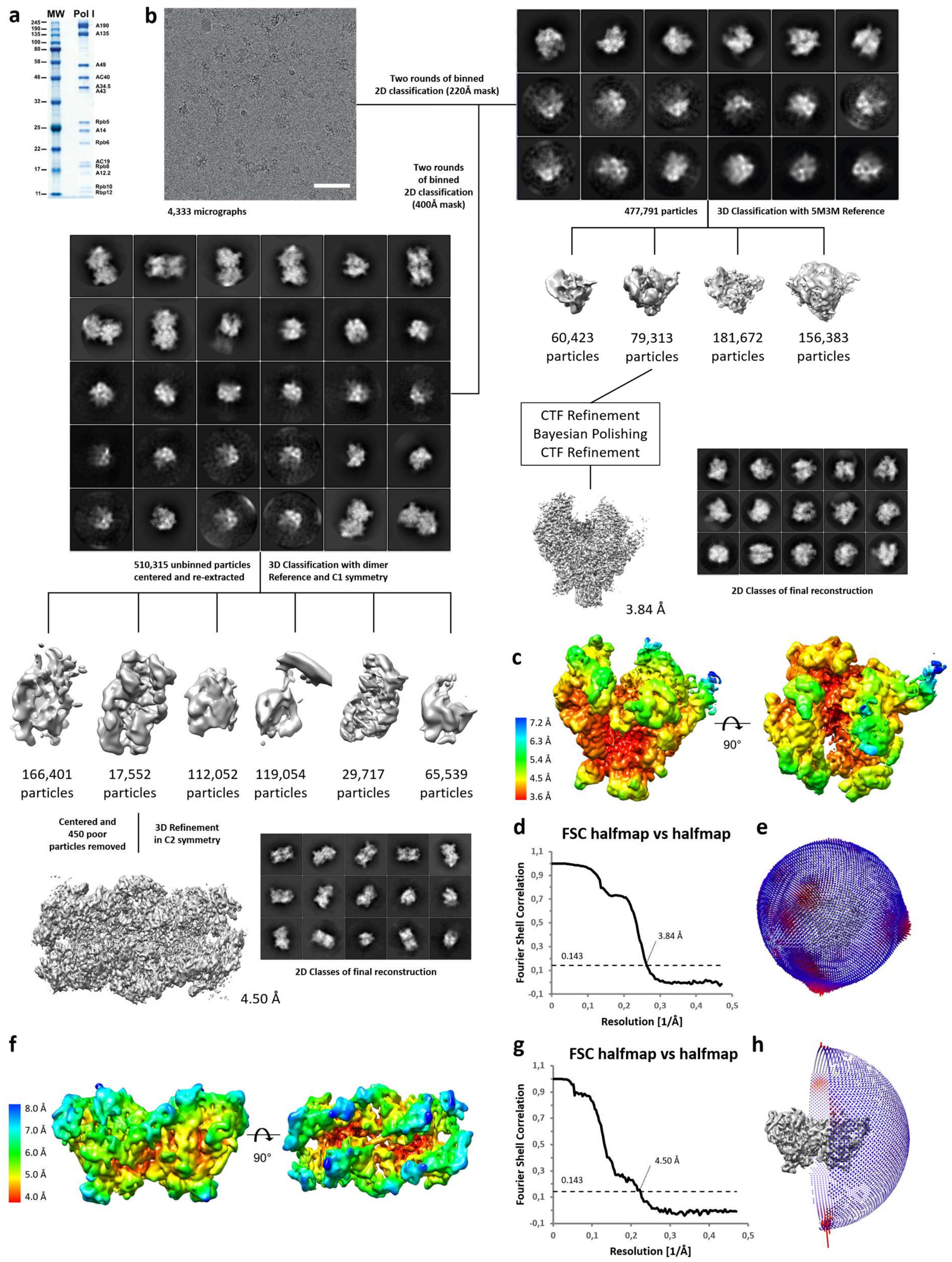

Supplementary Figure 1. Quality control and cryo-EM processing of *Sp* Pol I
a Coomassie-stained SDS-PAGE shows clear bands for all 14 subunits (confirmed by mass-spectrometry). MW: Molecular Weight marker. **b** Processing of cryo-EM data as described in the methods section. From one set of micrographs (scale bar: 50 nm), two sets of particles were extracted and separately analyzed yielding the reconstruction of a monomer in C1 symmetry and a dimer in C2 symmetry. Estimating from 3D classification, about 9.3% of particles appear to be in a dimeric state. **c** Local resolution of monomer reconstruction indicates flexibility of the stalk, jaw and clamp regions. (**d**) Fourier Shell Correlation (FSC) and (**e**) orientation distribution of Particles in the monomer reconstruction. Local Resolution (**f**), FSC (**g**) and orientation distribution (**h**) of dimers.

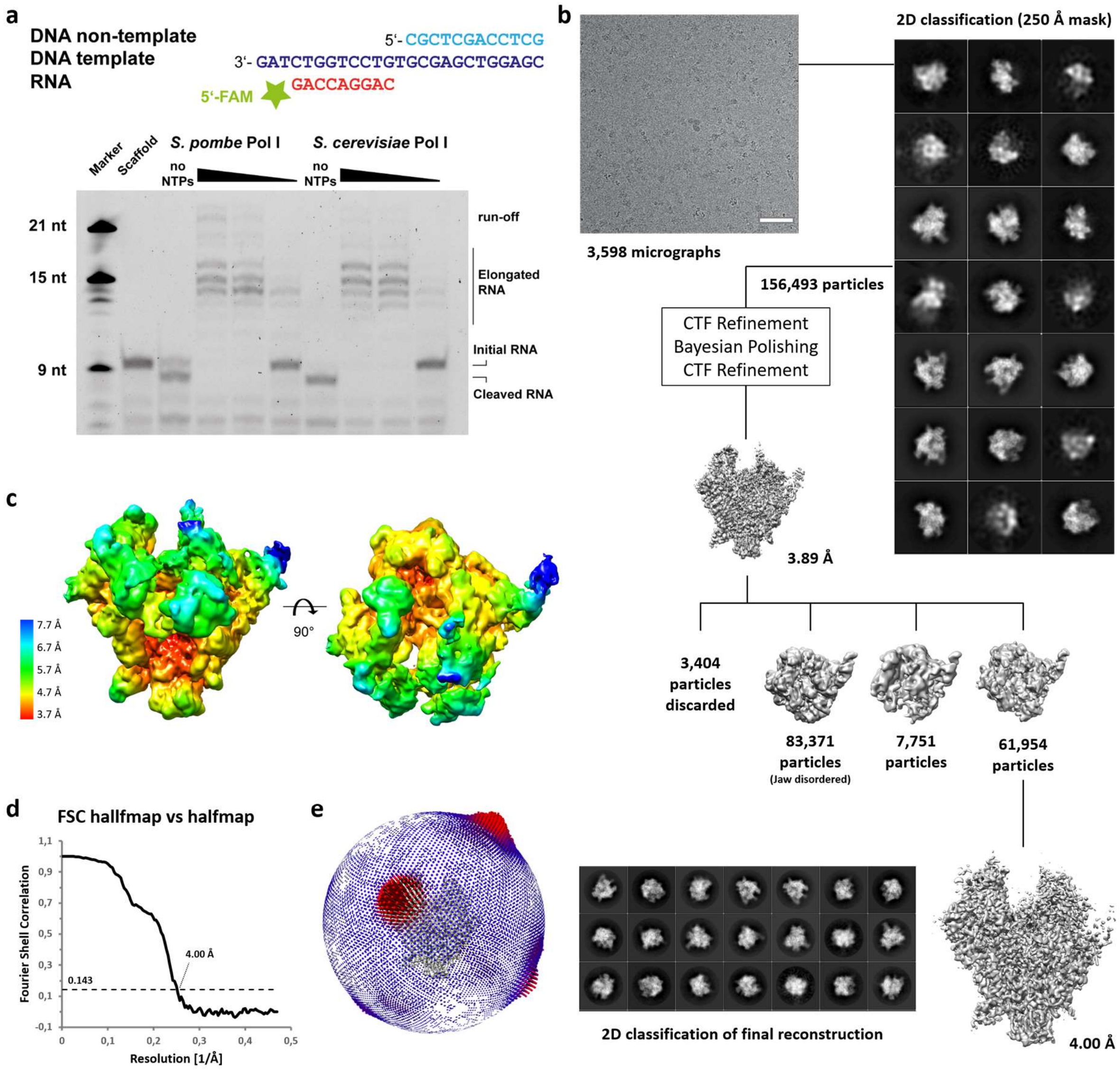

Supplementary Figure 2. *Sp* Pol I Elongation complex activity and cryo-EM processing
a Pol I purified from *Sp* is active in elongation and cleavage of an RNA primer *in vitro*. Efficiency is similar to its *Sc* homologue with an apparently slightly reduced cleavage efficiency. Top: representation of the used scaffold. Bottom: 20% urea PAGE (representative; FAM fluorescence readout; >30 individual reactions carried out in three separate experiments; details s. Methods). **b** Processing of EC cryo-EM data as described in the methods section. Scale bar: 50 nm. Nominal resolution of intermediate steps is higher compared to the final reconstructions. However, overall map quality improved by the removal of particles with highly flexible jaw and cleft regions. **c** Local resolution of EC reconstruction indicates flexibility of the stalk, jaw and clamp regions and the downstream DNA duplex. **d** Fourier Shell Correlation (FSC) of EC-halfmaps. **e** Orientation distribution of EC particles in the final reconstruction.

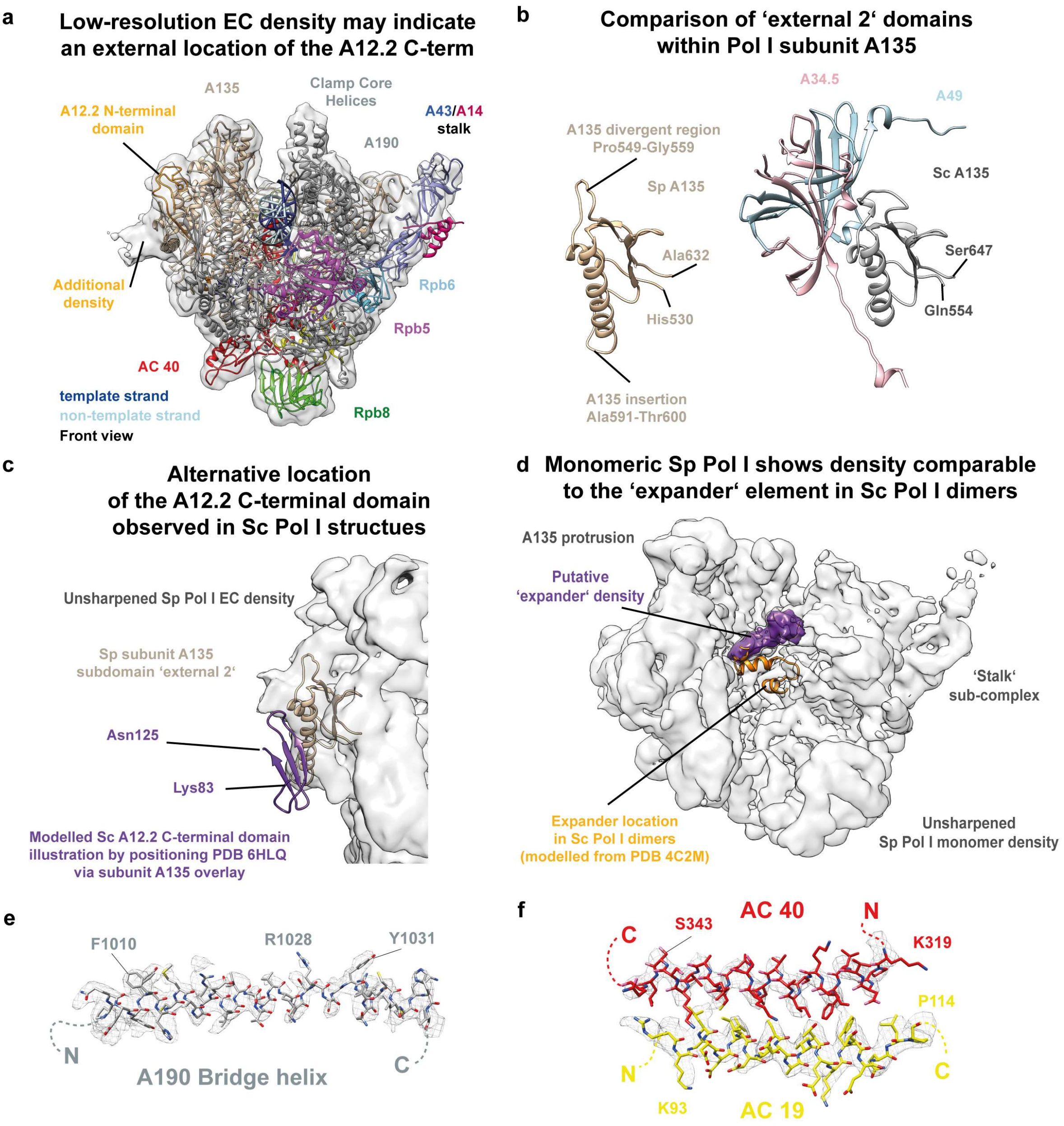

Supplementary Figure 3. Pol I monomers show similar features in *Sp* and *Sc*
a Low-resolution *Sp* EC density (unsharpened) may indicate the presence of the C-terminal domain of subunit A12.2 at a location on the outside of subunit A135. **b** The 'external 2' domains of subunit A135 diverge between *Sp* and *Sc*. **c** Unsharpened *Sp* Pol I EC density (grey; transparent) and 'external 2' domain model of subunit A135 (wheat). Location of the C-terminal domain of subunit A12.2 (violet) as observed in the 12-subunit *Sc* Pol I EC (PDB 6HLQ); modelled by overlay of subunit A135. **d** Unsharpened *Sp* Pol I monomer density (grey; transparent) with putative 'expander' density in violet. Ribbon model of the *Sc* expander as observed in the dimer (PDB 4C2M). Residues 1365-1391 of subunit A190 in orange (overlay via subunit A135). **e** Sharpened EC density (grey mesh) overlaid with the *Sp* Pol I EC bridge helix model (subunit A190) indicates residue orientations. *Sp* Arg1028 corresponds to Arg1015 in *Sc*. **f** Sharpened EC density (grey mesh) overlaid with the *Sp* Pol I EC models for subunit AC40 (red) and AC19 (yellow) indicates residue orientations and demonstrates the overall quality of the cryo-EM density.

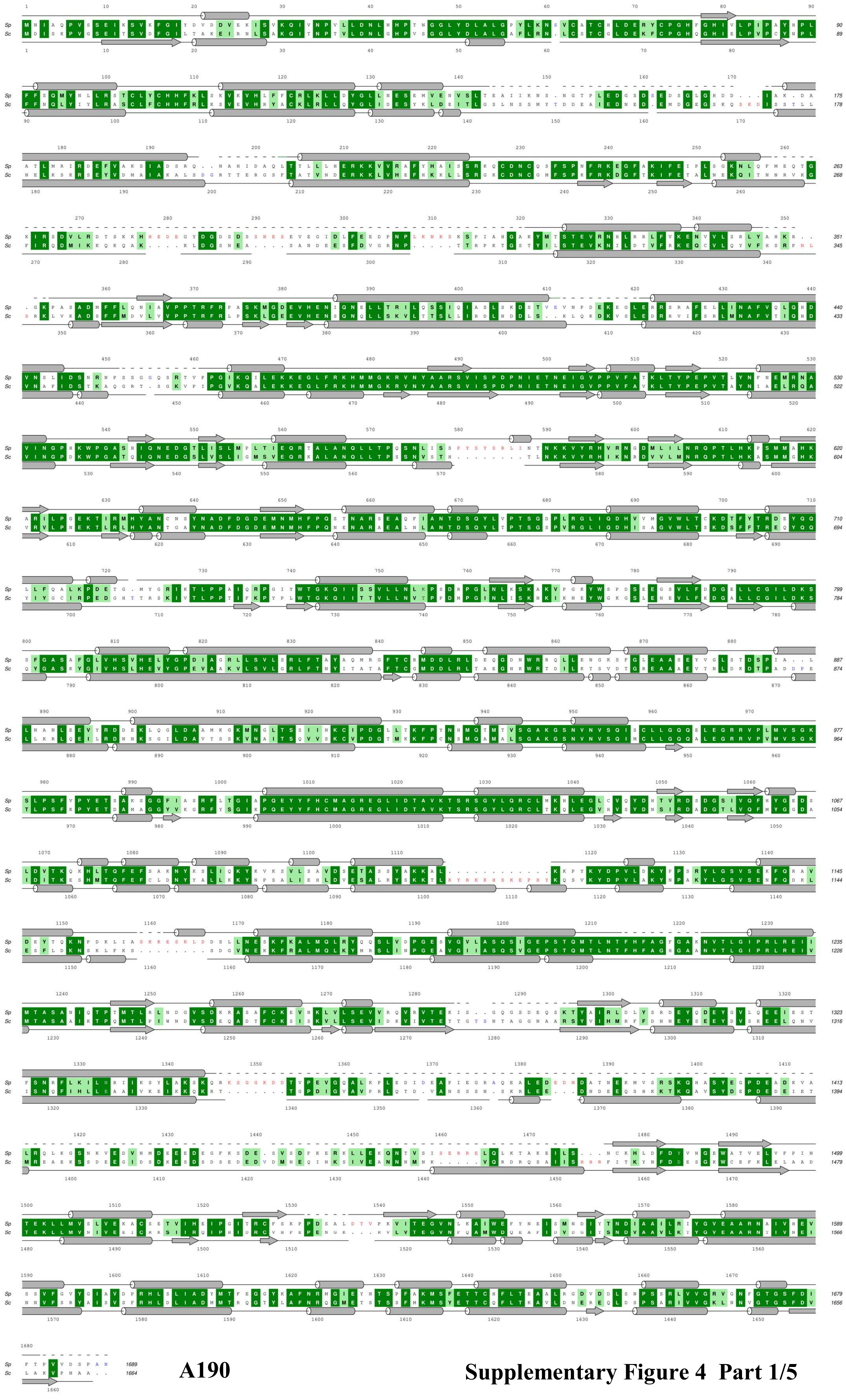

A190

Supplementary Figure 4 Part 1/5

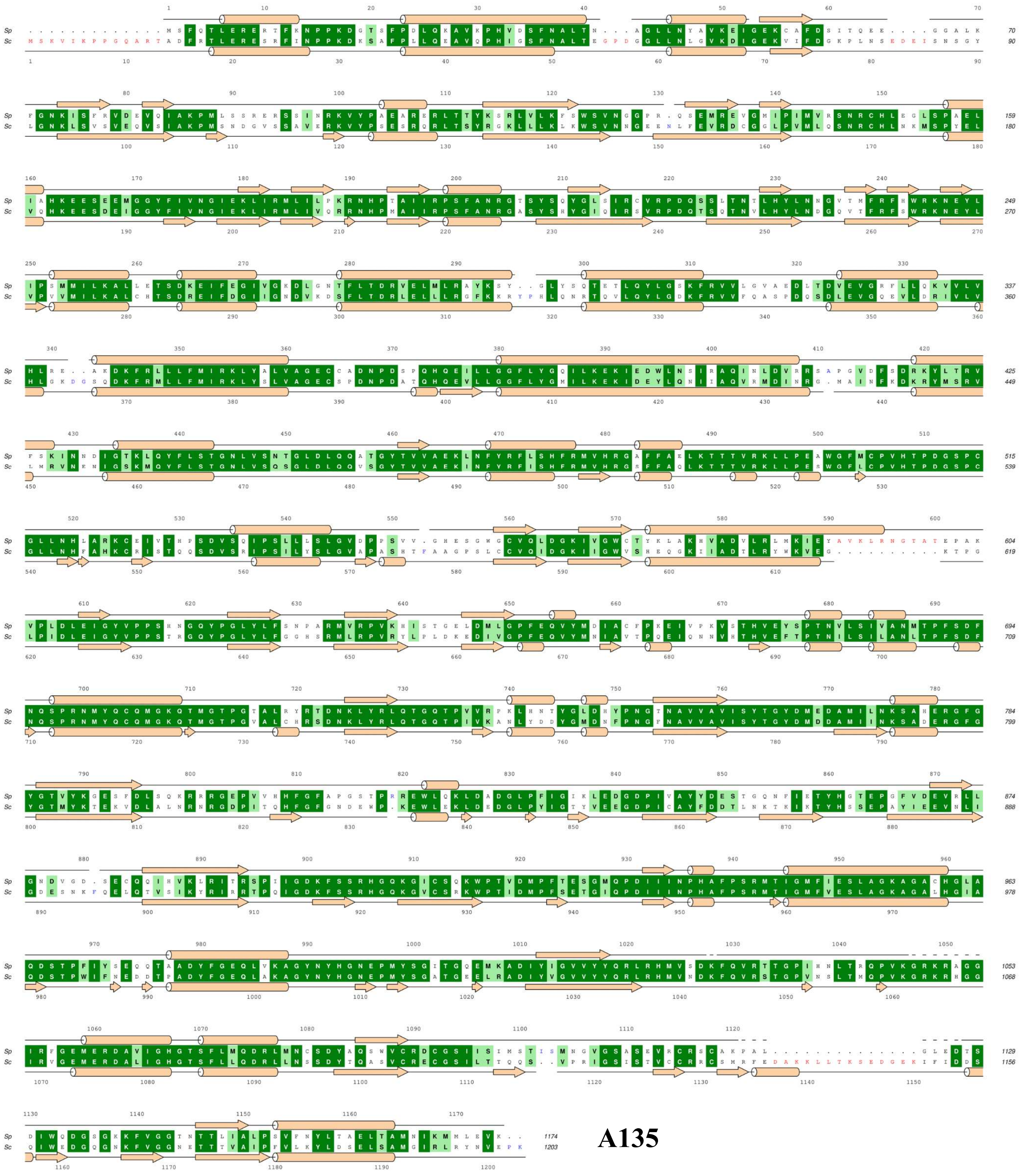

A135

Supplementary Figure 4 Part 2/5

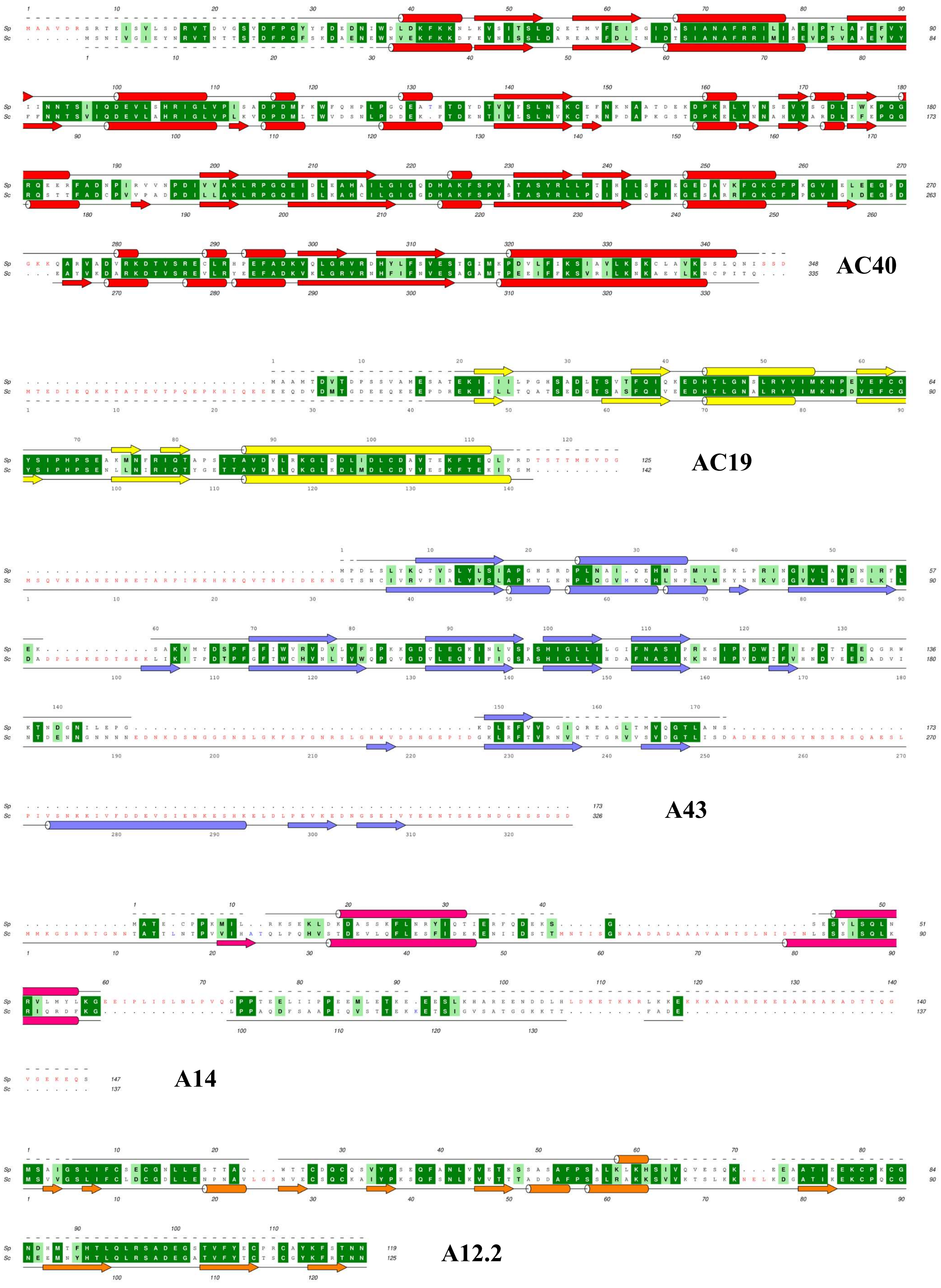

Supplementary Figure 4 Part 3/5

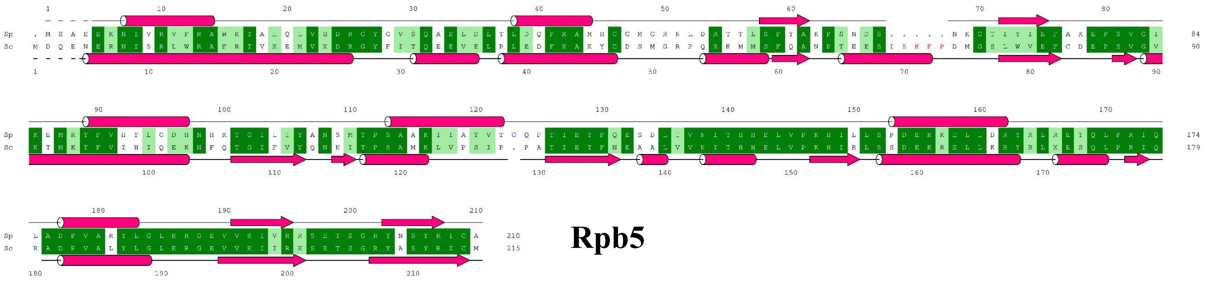

Rpb5

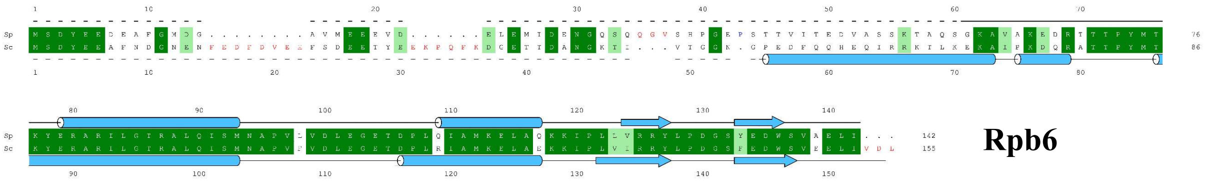

Rpb6

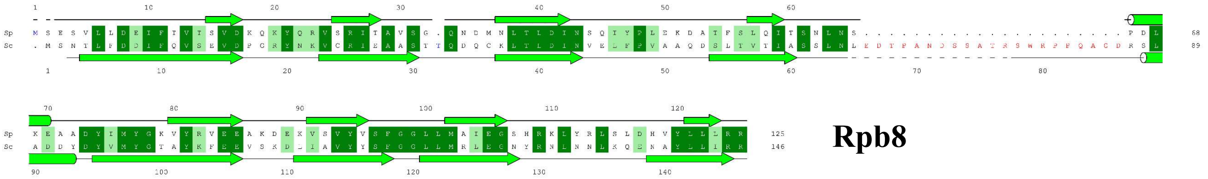

Rpb8

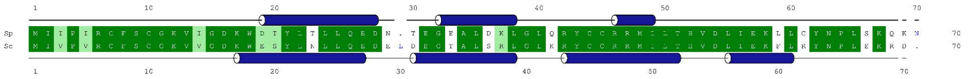

Rpb10

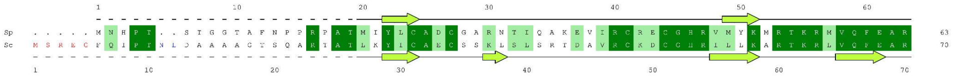

Rpb12

Supplementary Figure 4 Part 4/5

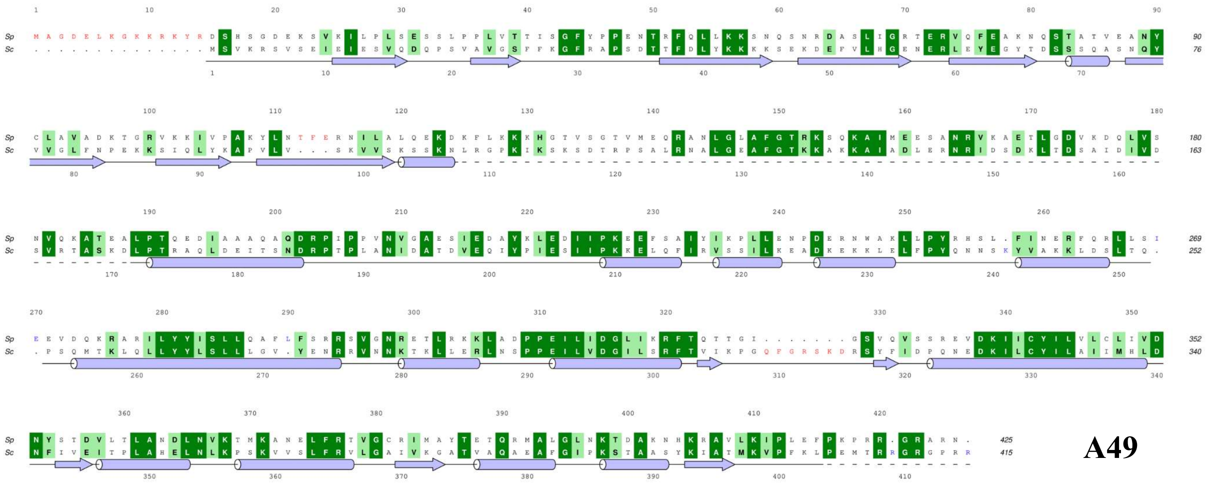

A49

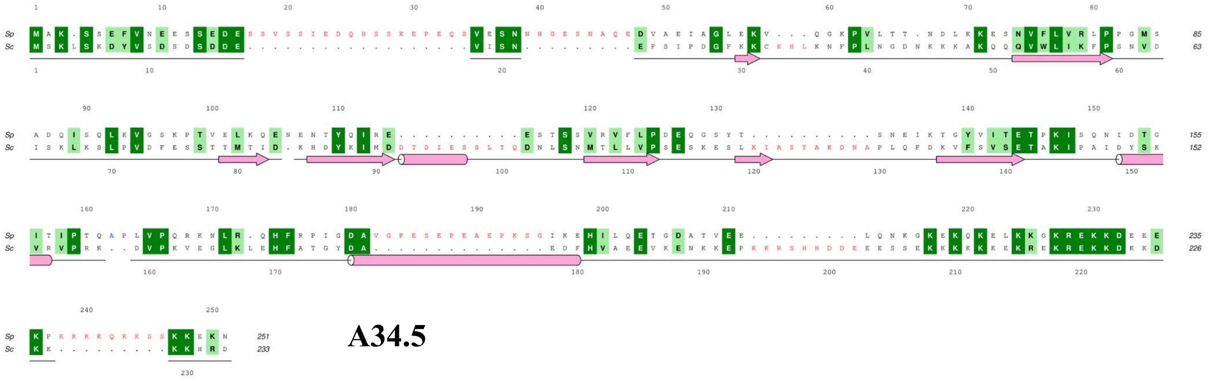

A34.5

Supplementary Figure 4 (Part 5/5). Structure-based sequence alignments of *Sp* and *Sc* Pol I subunits A190 (grey), A135 (wheat), AC40 (red), AC19 (yellow), A43 (slate), A14 (purple), A12.2 (orange), Rpb5 (pink), Rpb6 (blue), Rpb8 (green), Rpb10 (dark blue), Rpb12 (light green) and sequence alignment of the sub-complex A49 (light blue) / A34.5 (pink).
Identical amino acids are coded in dark green, conserved (DE, FWY, HKR, ILMV, NQ, ST, cutoff 0.500 in the program ALINE, s. Methods) amino acids in light green, insertions of less than three amino acids in blue letters, insertions of three or more residues in red.

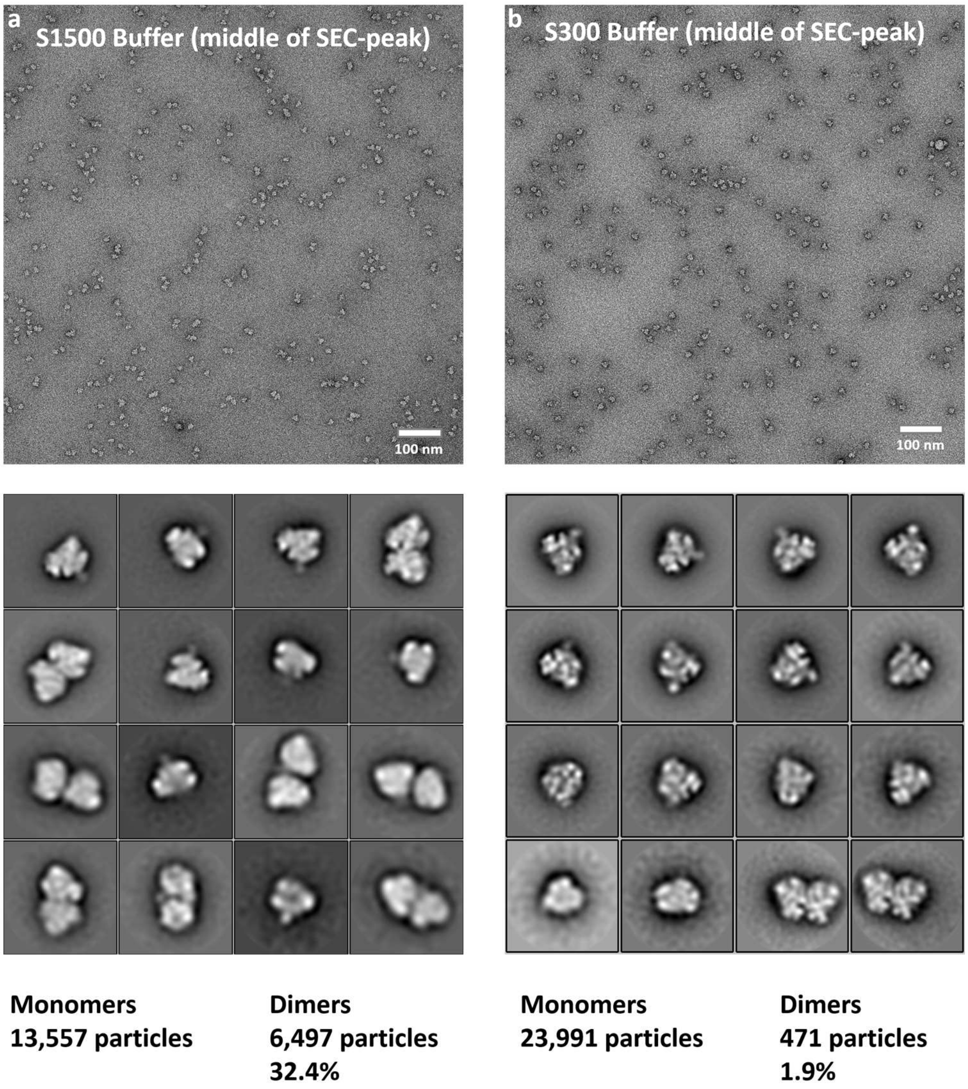

Supplementary Figure 5. Negative stain EM of non-crosslinked *Sp* Pol I. Peak fractions of analytical SEC show monomers and dimers independent of chemical crosslinking. The same sample was applied to high-salt (**a**) or low-salt (**b**) SEC (Fig. 2/Methods). Exemplary micrographs (top) show non-crowded single particles in monomeric or dimeric state. Particles were automatically picked and subjected to template-free 2D classification (junk particles removed). Particles in (**b**) were additionally centered in 3D due to dominant off-center picks. Quantification of 2D classes representing monomeric and dimeric particle states indicated a prevalence of 32.4% dimers in high-salt and 1.9% dimers in low-salt conditions.

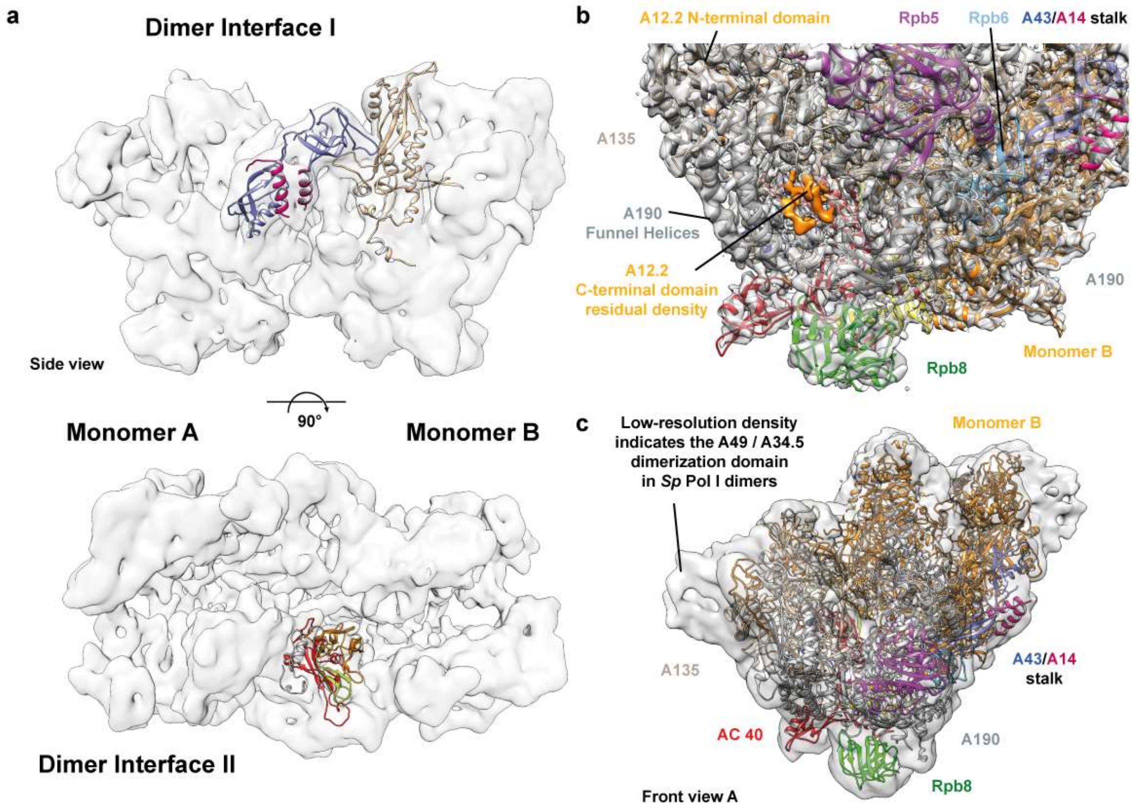

Supplementary Figure 6. The *Sp* Pol I dimer structure diverges from its *Sc* counterpart
a Pol I subunits involved in formation of interfaces I and II highlighted in the reconstruction of *Sp* Pol I dimers (unsharpened density as transparent envelop). **b** Sharpened cryo-EM density indicates the presence of the A12.2 C-terminal domain within the pore of *Sp* Pol I dimers. **c** Low-resolution density (unsharpened) may indicate the presence of the A49/A34.5 sub-complex in dimers. Likely, the sub-complex is either flexibly linked or sub-stoichiometrically attached in reconstructions.

Supplementary Table 1. Cryo-EM data collection, refinement and validation statistics.

	Monomeric *S.pombe* Pol I (EMDB-11840) (PDB 7AOC)	Dimeric form of *S.pombe* Pol I (EMDB-11841) (PDB 7AOD)	*S.pombe* Pol I Elongation Complex (EMDB-11842) (PDB 7AOE)
**Data collection and processing**			
Magnification	75k	75k	75k
Voltage (kV)	300	300	300
Electron exposure (e–/Å^2)	86	86	88
Defocus range (μm)	-1.4 - -2.4	-1.4 - -2.4	-1.8 - -2.8
Pixel size (Å)	1.0635	1.0635	1.0635
Symmetry imposed	C1	C2	C1
Initial particle images (no.)	477,791	510,315	156,493
Final particle images (no.)	79,313	17,102	61,954
Map resolution (Å)	3.84	4.50	4.00
FSC threshold	0.143	0.143	0.143
Map resolution range (Å)	3.6-7.2	4.0-9.0	3.7-6.7
**Refinement**			
Initial model used (PDB code)	5M3M	4C2M	5M3F
Model resolution (Å)	3.8	4.4	3.9
FSC threshold	0.143	0.143	0.143
Model composition			
Non-hydrogen atoms	29,725	59,258	30,829
Protein residues	3,746	7,466	3,768
Nucleotides	-	-	46
Ligands	6 (Zn)	12 (Zn)	6 (Zn)
B factors (Å^2)			
Protein	39.61	106.46	44.66
Nucleotides	-	-	142.12
Ligand	125.43	152.73	118.51
R.m.s. deviations			
Bond lengths (Å)	0.007	0.006	0.007
Bond angles (°)	0.871	0.884	0.916
Validation			
MolProbity score	2.29	2.72	2.42
Clashscore	15.63	26.98	21.52
Poor rotamers (%)	0.33	1.95	0.54
Ramachandran plot			
Favored (%)	88.50	88.95	88.30
Allowed (%)	11.23	10.73	11.38
Disallowed (%)	0.27	0.33	0.32

Supplementary Table 2. Sequence conservation of Pol I subunits.

Pol III subunit	Pol II subunit	Pol I subunit	Sequence Identity between *Sc* and *Sp* [%]
C160	Rpb1	A190	51.50
C128	Rpb2	A135	64.14
AC40	Rpb3	AC40	56.42
AC19	Rpb11	AC19	57.76
Rpb6	Rpb6	Rpb6	60.14
Rpb5	Rpb5	Rpb5	56.46
Rpb8	Rpb8	Rpb8	42.74
Rpb10	Rpb10	Rpb10	75.36
Rpb12	Rpb12	Rpb12	41.27
C17	Rpb4	A14	25.77
C25	Rpb7	A43	32.95
C11	Rpb9	A12.2	57.14
C53 (C4)	TFIIα (RAP74)	A49	29.14
C37 (C5)	TFIIβ (RAP30)	A34.5	26.26

Supplementary Table 3. Nucleic Acid Oligonucleotides used in this study

Sequence (5'->3')	Notes
AAGCTCAAGTACTTAAGCCTGGTCATTACTAGTACTGCC	DNA template for EC
GGCAGTACTAGTAAACTAGTATTGAAAGTACTTGAGCTT	DNA non-template for EC
UAUCUGCAUGUAGACCAGGC	LNA for EC
GATCTGGTCCTGTGCGAGCTGGAGC	DNA template for Elongation/Cleavage
GCTCCAGCTCGC	DNA non-template for Elongation/Cleavage
FAM-GACCAGGAC	FAM-labelled RNA for Elongation and marker
FAM-AACGGAGACCAGGAC	FAM-RNA marker
FAM-UGUUCUUCUGGAAGUCCAGTT	FAM-RNA marker
CCTAAAGGAGTCATCGAGCTTG	Genomic insertion primer fw
GCATCAATTAAAGGCGTCGC	Genomic insertion primer rev
CCTAAAGGAGTCATCGAGCTTGAAGAAGGACCTGATGGTAAAAAACAAGCCCGTGTAG<br>CAGACGTTCGAAAAGACACCGTATCCCGCGAATGCTTGAGACATCCCGAATTTGCAGA<br>TAAGGTGCAATTAGGCCGCGTACGCGATCATTATTTGTTTTCTGTTGAAAGCACCGGT<br>ATTATGAAGCCCGATGTCTTGTTCATTAAAAGCATTGCAGTCCTAAAATCGAAATGTC<br>TAGCCGTAAAGTCTAGTCTTCAGAATATTTCTTCTGACGATTACAAAGATGACGATGA<br>CAAGCATCACCATCACCATCACCATCACCATCACTGACAGCTGAAGCTTCGTACGCTG<br>CAGGTCGACGGATCCCCGGGTTAATTAAGGCGCGCCAGATCTGTTTAGCTTGCCTCGT<br>CCCCGCCGGGTCACCCGGCCAGCGACATGGAGGCCCAGAATACCCTCCTTGACAGTCT<br>TGACGTGCGCAGCTCAGGGGCATGATGTGACTGTCGCCCGTACATTTAGCCCATACAT<br>CCCCATGTATAATCATTTGCATCCATACATTTTGATGGCCGCACGGCGCGAAGCAAAA<br>ATTACGGCTCCTCGCTGCAGACCTGCGAGCAGGGAAACGCTCCCCTCACAGACGCGTT<br>GAATTGTCCCCACGCCGCGCCCCTGTAGAGAAATATAAAAGGTTAGGATTTGCCACTG<br>AGGTTCTTCTTTCATATACTTCCTTTTAAAATCTTGCTAGGATACAGTTCTCACATCA<br>CATCCGAACATAAACAACCATGGGTAAGGAAAAGACTCACGTTTCGAGGCCGCGATTA<br>AATTCCAACATGGATGCTGATTTATATGGGTATAAATGGGCTCGCGATAATGTCGGGC<br>AATCAGGTGCGACAATCTATCGATTGTATGGGAAGCCCGATGCGCCAGAGTTGTTTCT<br>GAAACATGGCAAAGGTAGCGTTGCCAATGATGTTACAGATGAGATGGTCAGACTAAAC<br>TGGCTGACGGAATTTATGCCTCTTCCGACCATCAAGCATTTTATCCGTACTCCTGATG<br>ATGCATGGTTACTCACCACTGCGATCCCCGGCAAAACAGCATTCCAGGTATTAGAAGA<br>ATATCCTGATTCAGGTGAAAATATTGTTGATGCGCTGGCAGTGTTCCTGCGCCGGTTG<br>CATTCGATTCCTGTTTGTAATTGTCCTTTTAACAGCGATCGCGTATTTCGTCTCGCTC<br>AGGCGCAATCACGAATGAATAACGGTTTGGTTGATGCGAGTGATTTTGATGACGAGCG<br>TAATGGCTGGCCTGTTGAACAAGTCTGGAAAGAAATGCATAAGCTTTTGCCATTCTCA<br>CCGGATTCAGTCGTCACTCATGGTGATTTCTCACTTGATAACCTTATTTTTGACGAGG<br>GGAAATTAATAGGTTGTATTGATGTTGGACGAGTCGGAATCGCAGACCGATACCAGGA<br>TCTTGCCATCCTATGGAACTGCCTCGGTGAGTTTTCTCCTTCATTACAGAAACGGCTT<br>TTTCAAAAATATGGTATTGATAATCCTGATATGAATAAATTGCAGTTTCATTTGATGC<br>TCGATGAGTTTTTCTAATCAGTACTGACAATAAAAAGATTCTTGTTTTCAAGAACTTG<br>TCATTTGTATAGTTTTTTTATATTGTAGTTGTTCTATTTTAATCAAATGTTAGCGTGA<br>TTTATATTTTTTTTCGCCTCGACATCATCTGCCCAGATGCGAAGTTAAGTGCGCAGAA<br>AGTAATATCATGCGTCAATCGTATGTGAATGCTGGTCGCTATACTGCTGTCGATTCGA<br>TACTAACGCCGCCATCCAGTTTAAACGAGCTCGAATTCATCGATGATATCAGATCCAC<br>TAGTGGCCTATGCATGTCTGTTAAATTAGTTTTATATGTAATCTTTTGGATAATTGGG<br>TACGCTGTTAAAAAATATACTTTTGAAAGTTATTGATTTCTATATTTCAATATTAACA<br>TTTTGTCCTTTCTTTAATTTTAAATTTAATTCATCATAGACATGTATTTTGTTGATAA<br>CCAGTAGACCTTTTAAAAAGAAGTGTTGGTTTTCAATCACTACTTGCCATCCTTTGAA<br>AGTCCTTATAAAATGCTTTGAGAATTTATATGAAAGCGACGCCTTTAATTGATGC	Synthetic construct for genomic insertion of FLAG/HIS-tag
