# Supplementary material for: Conserved strategies of RNA polymerase I hibernation and activation
Source: Nat Commun. 2021 Feb 3;12:758. doi: 10.1038/s41467-021-21031-8 (PMC7859239; doi:10.1038/s41467-021-21031-8)
Supplement: Supplementary file 3 — Description of Additional Supplementary Files [file 41467_2021_21031_MOESM3_ESM.pdf]

Description of Additional Supplementary Files

Supplementary Movie 1. Expansion of the active center cleft in *Sp* Pol I. The structure of a *Sp* Pol I EC rotated in X and Y orientation starting from canonical front view. Morph (Molecular dynamics interpolation) to monomer and dimer conformation (reversed and repeated once) illustrates the expansion of the active center cleft. Finally, the second monomer in *Sp* Pol I dimers is faded in.

Supplementary Movie 2. Comparison of Pol I dimer architecture in *Sp* and *Sc*. The structure of a *Sp* Pol I dimer is rotated in X and Y orientation starting from canonical front view of monomer A. Morph (Molecular dynamics interpolation) to the *Sc* Pol I dimer conformation illustrates the differences in architecture, especially regarding stalk-interaction with monomer B and further cleft expansion in *Sc*. Followed by reverse morph to *Sp* orientation, and repeated morph to *Sc* conformation from side view of dimers.
